# Revision rates and progression to shoulder arthroplasty after arthroscopic repair of massive rotator cuff tears

**DOI:** 10.1002/ksa.12651

**Published:** 2025-04-01

**Authors:** Umile Giuseppe Longo, Alberto Lalli, Benedetta Bandini, Alice Piccolomini, Nathan S. Ullman, Andrea Vaiano, Pieter D'Hooghe

**Affiliations:** ^1^ Fondazione Policlinico Universitario Campus Bio‐Medico Rome Italy; ^2^ Department of Statistical Sciences Sapienza University of Rome Rome Italy; ^3^ Centre de Recherches en Mathématiques de la Décision, CNRS Université Paris‐Dauphine, PSL University Paris France; ^4^ Aspetar Orthopedic and Sports Medicine Hospital, Aspire Zone Doha Qatar

**Keywords:** arthroscopy, massive irreparable rotator cuff tear, partial repair, revision reverse total shoulder arthroplasty, superior capsular reconstruction

## Abstract

**Purpose:**

The purpose of this systematic review was to assess the rate of progression to reverse total shoulder arthroplasty (RTSA) and to other interventions as revision surgeries after an arthroscopic repair of a massive rotator cuff tear (MRCT). Additionally, the review aimed at defining the best arthroscopic approach for the treatment of MRCTs in terms of failure and revision rates.

**Methods:**

The purpose of this systematic review and meta‐analysis was to evaluate the rates of progression to reverse total shoulder arthroplasty in patients who underwent primary arthroscopic repair of an MRCT with different arthroscopic procedures. A meta‐analysis was performed to compare the rate of progression to revision surgery and reverse total shoulder arthroplasty.

**Results:**

Eighteen articles were included in the qualitative synthesis and 14 articles were included in the meta‐analysis. Overall, 934 patients and 950 shoulders were involved in the review. Seven‐hundred and thirty patients and 735 shoulders were included in the meta‐analysis. The proportion of revisions to reverse total shoulder arthroplasty was 0.9%, 3.3% and 0.1% for complete repair, partial repair and superior capsular reconstruction, respectively. No statistically significant differences were found across the groups in terms of progression to reverse total shoulder arthroplasty (n.s.). The average proportions of revisions to interventions different than reverse total shoulder arthroplasty. were 0.9% for complete repair, 2.0% for partial repair and 2.0% for superior capsular reconstruction again, no statistically relevant difference was found among the groups (n.s.).

**Conclusions:**

The current review finds no statistically significant differences in the progression to reverse total shoulder arthroplasty or other revision procedures among partial repair, complete repair and superior capsular reconstruction for massive irreparable rotator cuff tears. It is crucial to understand the long‐term outcomes of different surgical techniques for massive rotator cuff tears, particularly regarding failure rates and progression to further procedures.

**Level of Evidence:**

Level IV.

**Abbreviations**
LOE: level of evidence
MRCT: massive rotator cuff tear
PICOS: patient, intervention, comparison, outcome and study design
PRISMA: Preferred Reporting Items for Systematic Reviews and Meta‐analyses
RCT: randomised control trials
ROBINS‐I: risk of bias in non‐randomised studies of interventions
RoB 2: risk of bias tool for randomised trials
RTSA: reverse total shoulder arthroplasty
SD: standard deviation

## INTRODUCTION

Massive tears of the tendons of the rotator cuff cause hypotrophy and fatty degeneration of the rotator cuff muscles and painful loss of function of the shoulder [20, 34, 44]. The overall incidence of rotator cuff tears ranges from 5% to 40% [2], with approximately 54% of individuals over the age of 60 having a partial or complete rotator cuff tear [28, 32].

Numerous treatment strategies have been proposed to treat irreparable massive rotator cuff tears and when conservative treatments fail, or in trauma cases, surgical treatment should be considered [4, 17]. There is no consensus when it comes to the treatment of large or massive rotator cuff tears [7, 53].

An arthroscopic approach to managing pseudo paralysis may be desirable, if it can reliably restore function while avoiding the morbidity of a joint replacement, particularly reverse total shoulder arthroplasty [3, 11]. These interventions have shown a high success rate of subjective and functional results [35, 45]. However, persistent tears or re‐tears after rotator cuff repair have been seen in up to 35% of small tears and in as high as 94% of larger multi‐tendon tears [31, 40]. Structural failure rates have been reported to range from 20% to 94%, and the failure usually occurs at the tendon‐bone interface [37, 51].

For this reason, indications for reverse total shoulder arthroplasty have continued to expand, including the use of reverse total shoulder arthroplasty as a revision procedure for failed shoulder surgery [5], even though revision surgery for failed rotator cuff repair is fraught with intraoperative challenges such as loss of elasticity, atrophy and retraction of the remaining tendon [16, 41]. In the current literature, there is a lack of evidence on whether the primary approach to massive rotator cuff tears should be performed arthroscopically or directly via reverse total shoulder arthroplasty.

Also, it is yet to be established whether one arthroscopic repair technique is superior the others. For this reason, the purpose of this systematic review was to assess the rate of progression to reverse total shoulder arthroplasty and to other revision procedures after an arthroscopic repair of a massive rotator cuff tears. Additionally, the review aimed at defining the best arthroscopic approach for the treatment of massive rotator cuff tears in terms of failure and revision rates by comparing the complete repair, the partial repair and the superior capsular reconstruction techniques. The hypothesis of this study was that primary arthroscopic repairs of massive rotator cuff tears would result in low failure rates and a low rate of progression to reverse total shoulder arthroplasty.

## MATERIALS AND METHODS

### Search strategy

A systematic review was performed using the Preferred Reporting Items for Systematic Reviews and Meta‐analyses (PRISMA) guidelines [43] Medline, EMBASE, Scopus, CINAHL and CENTRAL bibliographic databases were searched using the following string: ((((massive) OR (irreparable)) AND (rotator cuff)) AND (((injury) OR (tear)) OR (lesion))) AND (shoulder replacement). Articles from inception to November 2023 were searched.

### Study screening

Data extraction was performed by two independent reviewers and differences were reconciled by mutual agreement. In case of disagreement for inclusion/exclusion of articles, the consensus of a third reviewer was asked.

**Figure 1:**
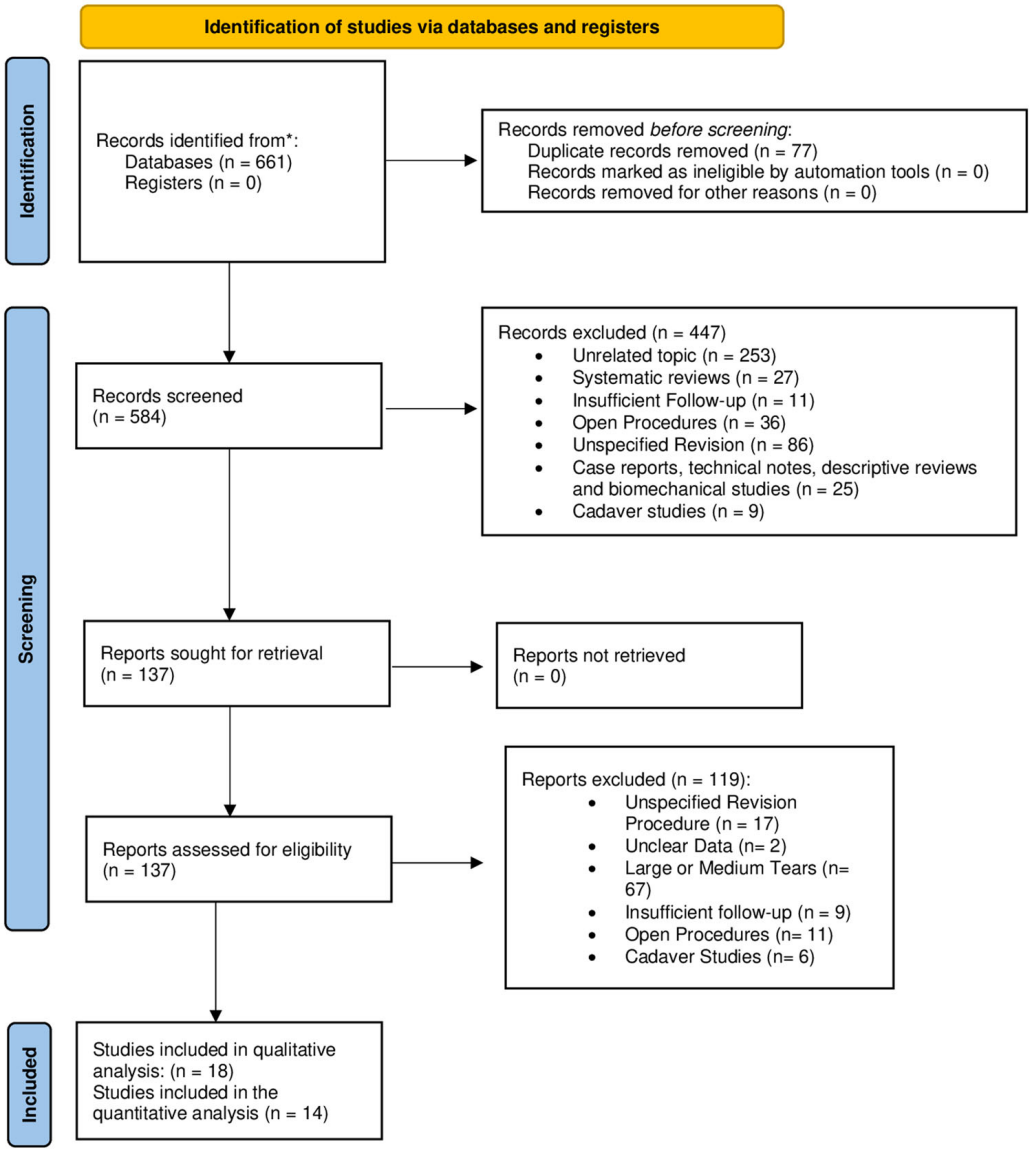
PRISMA flow diagram for identification of studies via databases and registers.

The number of articles included or excluded were registered and reported in the PRISMA flowchart (Figure 1).

### Data extraction

The research question was formulated using a PICOS‐approach: Patient (P); Intervention (I); Comparison (C); Outcome (O) and Study design (S). The purpose of this systematic review and meta‐analysis was to evaluate the rates of progression to reverse total shoulder arthroplasty in patients who underwent primary arthroscopic repair of a massive rotator cuff tears with different arthroscopic procedures.

General study characteristics extracted were author, year of publication, type of study, level of evidence (LOE), sample size, age, gender, number of shoulders treated and pre‐operative shoulder arthritis grades. Moreover, the type of arthroscopic procedure performed, and follow‐up were reported (in case of multiple measurements, the one at last follow‐up was considered) (Table 1).

**Table 1:** Demographics.

Author; year	Intervention	*N* of patients	*N* of shoulders	Mean age (range (years))	Gender (M:F)
Chillemi; 2021	SCR LHB graft	25	25	61.4 (39–76)	17:8
Chung; 2013	Complete repair	108	108	63.7 ± 6.4	44:64
Denard; 2012	Complete repair, Partial repair	115	126	62.3 (37–81)	74:41
Farazdaghi; 2020	Complete repair, Partial repair	27	27	63.7 (41–87)	17:10
Galasso; 2016	Partial repair	90	95	62.7 ± 7.3 (40–76)	41:49
Hallock; 2020	Partial repair	38	38	66 (45‐79)	24:14
Heuberer; 2015	Debridement	23	23	66.5 ± 6.9	11:12
	Partial repair	22	22	68.0 ± 8.0	14:8
	Complete repair	23	23	65.0 ± 6.8	15:8
Jung; 2022	Partial repair	63	63	65.3 (51–79)	40:23
Kim; 2021	Partial repair	57	57	Group 1: 62.9 ± 7.0 (51–73) Group 2: 63.7 ± 6.3 (55–72)	22:35
Lacheta; 2020	SCR Dermal Allograft	22	22	57 (41–65)	13:9
Leow; 2020	Complete repair	42	42	59.7 ± 8.1	19:23
Ma; 2019	Complete repair	71	71	65.2 ± 7.5 (44–83)	24:47
Malahias; 2019	Complete repair	34	34	67.2 ± 8.6	39:25
	Partial repair	30	30		
Senekovic; 2012	Subacromial Balloon Spacer	20	20	70.5 (54–85)	11:9
Takayama; 2021	SCR TLF graft	54	54	Group 1: 72.8 ± 3.5 Group 2: 69.2 ± 5.3	30:24
Van der Zwaal; 2012	Complete repair	31	31	59 ± 4.7	15:16
Vogler; 2020	Debridement	19	19	68 (55–80)	9:10
Yang 2021	Complete repair	20	20	Group 1: 59.9 ± 6.5 Group 2: 61.5 ± 6.1	11:9

Abbreviations: F, females; LHB, long head of the biceps; M, males; N, number; SCR, superior capsular reconstruction; TNF, tensor fascia lata.

Outcome measures extracted included: post‐operative progression to reverse total shoulder arthroplasties after the arthroscopic repair, indications for reverse total shoulder arthroplasty and time of reverse total shoulder arthroplasty (Table 2).

**Table 2:** Progression to RTSA.

Author; year	Intervention	Mean follow‐up (range (months))	Revisions to RTSA	Indications for RTSA (*N*)	Time to RTSA (months)
Chillemi; 2021	SCR LHB graft	Min 12	0	0	0
Chung; 2013	Complete repair	31.7 ± 15.8 (14–71)	0	0	0
Denard; 2011	Complete repair, partial repair	98.9 (62–149)	0	0	0
Farazdaghi; 2020	Complete repair, partial repair	94.8 (min 60)	2	NR	NR
Galasso; 2016	Partial repair	82.7 ± 37.1 (24–148)	4	Unsatisfactory recovery	NR
Hallock; 2020	Partial repair	54 (28.8–75.6)	1	Poor functional recovery and pain	25
Heuberer; 2015	Debridement	42 (23–70)	1	Loss of function and persistent pain	NR
	Partial repair	42 (23–70)	0	0	0
	Complete repair	42 (23–70)	1	Loss of function and persistent pain	NR
Jung; 2022	Partial repair	30.2 (24–80)	3	NR	NR
Kim; 2021	Partial repair	Min 60	2	Pain and disability with progressing arthropathy	60, 84
Lacheta; 2020	SCR dermal allograft	25 (24–36)	0	0	0
Leow; 2020	Complete repair	17.5 (min 12)	0	0	0
Ma; 2019	Complete repair	24 (24–24)	0	0	0
Malahias; 2019	Complete repair	32.7 ± 29.5	0	0	0
	Partial repair		0		
Senekovic; 2012	Subacromial balloon spacer	36 (36–36)	1	Unsatisfactory recovery	1.5
Takayama; 2021	SCR TLF graft	42 (24–74)	1	Loss of function	27
Van der Zwaal; 2012	Complete repair	26.5 ± 2.3	1	Symptomatic re‐rupture	NR
Vogler; 2020	Debridement	47 (28–62)	5	Rotator cuff arthropathy	63 (45–97)
Yang 2021	Complete repair	(12–24)	1	Severe cuff retraction and fatty infiltration	NR

Abbreviations: LHB, long head of the biceps graft; Min, minimum; *N*, number; NR, not reported; RTSA, reverse total shoulder arthroplasty; SCR, superior capsular reconstruction; TNF, tensor fascia lata.

Also, the progression and timing to revision surgeries other than reverse total shoulder arthroplasty were also reported, with the corresponding indications (Table 3).

**Table 3:** Other revisions.

Author; year	Intervention	Revisions other than RTSA (*N*)	Indications for Revision (*N*)	Months to revision other than RTSA
Chillemi; 2021	SCR LHB graft	Capsular release (1)	Stiffness (1)	6
Chung; 2013	Complete repair	0	0	0
Denard; 2011	Complete repair, Partial repair	Capsular release (3) Revision repair (1) Closed reduction (1)	Stiffness (3) Traumatic event (2)	NR
Farazdaghi; 2020	Complete repair, Partial repair	SCR (4)	NR	NR
Galasso; 2016	Partial repair	Latissimus Dorsi tendon transfer (2)	Unsatisfactory Recovery (2)	61.6 ± 34.3 (24–121)
Hallock; 2020	Partial repair	Revision repair (1)	Acute retear after fall (1)	0.5
Heuberer; 2015	Debridement	Open lavage and debridement (2)	Infection (2)	NR
	Partial repair	Anchor removal (1)	Implant loosening (1)	
	Complete repair	Open lavage and debridement (1)	Infection (1)	
Jung; 2022	Partial repair	NR (2)	NR	NR
Kim; 2021	Partial repair	0	0	0
Lacheta; 2020	SCR dermal allograft	Revision SCR (1)	Loss of function	14.4
Leow; 2020	Complete repair	Open lavage and debridement (1)	Infection	NR
Ma; 2019	Complete repair	0	0	0
Malahias; 2019	Complete repair	Debridement (1)	Persistent pain and weakness (1)	NR
	Partial repair	0	0	0
Senekovic; 2012	Subacromial Balloon Spacer	0	0	0
Takayama; 2021	SCR TLF graft	0	0	0
Van der Zwaal; 2012	Complete repair	0	0	0
Vogler; 2020	Debridement	0	0	0
Yang 2021	Complete repair	0	0	0

Abbreviations: LHB, long head of the biceps graft; Min, minimum; *N*, number; NR, not reported; RTSA, reverse total shoulder arthroplasty; SCR, superior capsular reconstruction; TNF, tensor fascia lata.

### Inclusion criteria

The scope encompassed studies detailing patients undergoing primary arthroscopic repair for a massive rotator cuff tear. Eligible studies further required explicit specification of the type of arthroscopic procedure performed. A massive rotator cuff tear was defined as a tear of at least 5 cm or involvement of two or more tendons of the rotator cuff. Additionally, a minimum mean follow‐up duration of 12 months was mandated.

### Exclusion criteria

Technical notes, letters to editors, instructional courses or studies involving procedures other than reverse shoulder arthroplasty were systematically excluded. In vitro, animal, cadaver, and biomechanical studies were also excluded from consideration. Studies failing to specify the type of arthroscopic procedure performed were not incorporated. Furthermore, studies reporting outcomes for patients with pre‐operative shoulder osteoarthritis of Grades 4–5 according to the Hamada classification, irreparable subscapularis tears, or medium‐large rotator cuff tears were systematically discarded. Studies with a minimum follow‐up duration of less than 12 months were not included. Those involving patients undergoing a revision rotator cuff repair were deemed ineligible. Studies lacking clear reporting on revision surgery rates, follow‐up, or presenting unclear or missing data were likewise excluded from the current review.

### Quality assessment

Given the designs of the included studies, the Risk of Bias in Non‐Randomised Studies of Interventions (ROBINS‐I) tool, the Risk of Bias (RoB 2) tool for Randomised Trials by Cochrane and the Joanna Briggs Institute Critical Appraisal Tool for Case series, were used to assess the quality of each study. No RCTs met the eligibility criteria [48, 49].

### Statistical analysis

Categorical data were recorded in the form of frequencies and recoded as percentages. Wilson confidence intervals for proportions were computed when necessary. This is recommended when dealing with small *n* or extreme proportions and is a common practice in biostatistics. Continuous data were recorded as mean ± standard deviation (SD). Since no reasonable probabilistic assumptions can be made on the data, the non‐parametric Kruskal–Wallis rank sum test was implemented to compare groups. In order to verify the statistical significance, a Kruskal–Wallis test was performed. The null hypothesis is that the samples share the same mean: explicitly that the three interventions lead to the same average number of reverse total shoulder arthroplasty. The alternative hypothesis is that the means differ for at least one of the interventions.

If the null hypothesis is rejected, further testing procedures can be implemented to perform a pairwise comparison between the samples. A *p*‐value lower than 0.05 was set as the significance level for the test. All the analyses were performed in R (R Foundation for Statistical Computing, https://www.r-project.org/).

## RESULTS

### Study selection

Six hundred and sixty‐one articles were identified after the literature search. No additional studies were found in the grey literature and no unpublished articles were retrieved. Five‐hundred and eighty‐four articles were left after duplicate removal.

Four‐hundred and forty‐seven articles were excluded based on title and abstract (unrelated topic *n* = 253; systematic reviews *n* = 27; insufficient follow‐up, *n* = 11; open procedures *n* = 36; no type of revision surgery specified *n* = 86; case reports, technical notes, biomechanical studies, simulations, *n* = 25; studies on cadavers, animals, simulations, biomechanical, histological or in vitro studies *n* = 9).

One‐hundred and thirty‐seven articles were reviewed by full text and 119 of those were excluded (no type of revision surgery specified *n* = 17; studies with unclear data *n* = 2; studies including patients with large or medium rotator cuff tears, *n* = 67; insufficient follow‐up *n* = 9; open procedures 11; cadaveric studies *n* = 6; no full text available *n* = 7).

At final screening, 18 articles met the selection criteria and were included in the review [8, 9, 10, 14, 18, 22, 23, 25, 26, 29, 30, 36, 38, 46, 50, 52, 54, 55]. Fourteen articles were included in the meta‐analysis [8, 9, 18, 22, 23, 25, 26, 29, 30, 36, 38, 50, 52, 55], according to stratification criteria.

No articles were excluded following the risk of bias assessment. The PRISMA flowchart of the literature search is reported in Figure 1.

### Population demographics

Overall, 934 patients and 950 shoulders were involved in the review. However, due to the lack of studies reporting data on other techniques, it was only possible to compare the following techniques in the quantitative analysis: complete repair, partial repair and superior capsular reconstruction. This resulted in a total of 730 patients and 735 shoulders being included in the meta‐analysis. Three‐hundred and twenty‐nine patients and shoulders were treated with a complete repair. Three‐hundred patients involving 305 shoulders received a partial repair. One‐hundred and one patients and shoulders received superior capsular reconstruction with either a dermal allograft, an autologous graft from the tendon fascia lata or with the long head of the biceps tendon as an augmentation technique.

### Quality of evidence

The only RCT was judged as having low risk of bias [36]. Five retrospective case–control studies were judged as having low risk of bias [23, 25, 26, 29, 50], while five other retrospective case‐controls had a moderate risk of bias [10, 14, 30, 38, 55]. RCSs studies were overall of good quality [8, 9, 18, 22, 46, 52, 54].

**Figure 2:**
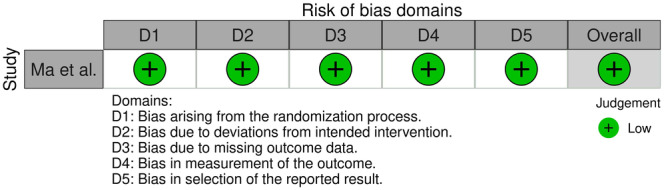
Risk of bias assessment via risk of bias tool for randomised trials (RoB 2).

**Figure 3:**
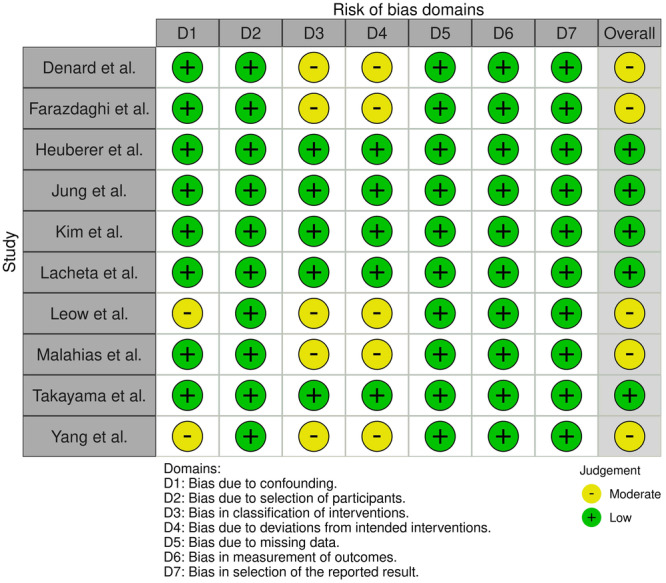
Risk of Bias assessment via risk of bias in non‐randomised studies of interventions (Robins‐I).

**Figure 4:**
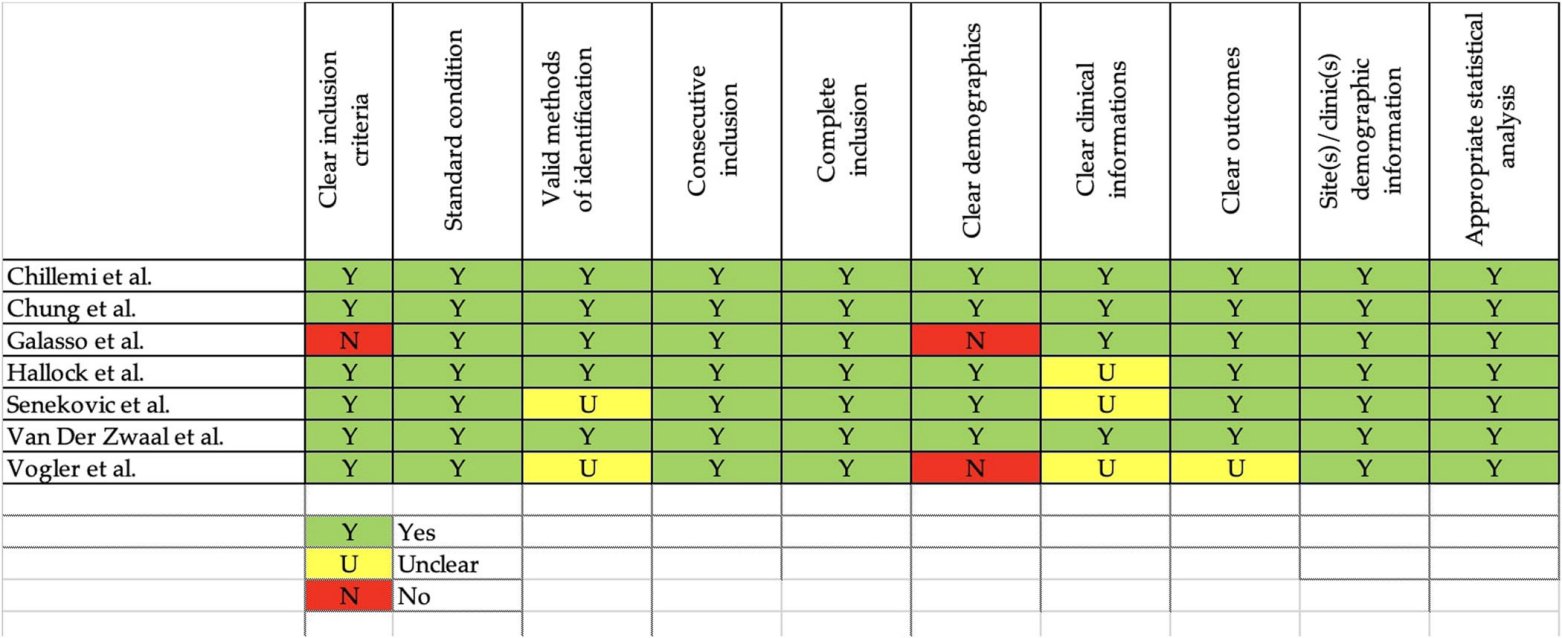
Risk of bias assessment via Joanna Briggs institute critical appraisal tool for CSs.

The risk of bias assessment is reported in Figures 2, 3, 4.

### Meta‐analysis results

Sixteen studies were included in the meta‐analysis, divided in three groups of intervention: complete repair, partial repair and superior capsular reconstruction. Other interventions were not considered due to the insufficient number of patients included in those cohorts.

Seven‐hundred and thirty patients and 735 shoulders were considered. From these population, there were 14 revisions to reverse total shoulder arthroplasty and 11 revisions to interventions other than reverse total shoulder arthroplasty. Only three studies did not lead to any kind of revision [9, 36, 38].

Table 4 summarises counts and proportions of revisions for the studies considered in the meta‐analysis.

**Table 4:** Progression to revision surgery per each study.

Author; year	Intervention	*N* of Patients	*N* of Shoulders	Revisions to RTSA	% RTSA	Revisions no RTSA	% no RTSA
Chillemi; 2021	SCR	25	25	0	0.00	1	4.00
Chung; 2013	Complete repair	108	108	0	0.00	0	0.00
Galasso; 2016	Partial repair	90	95	4	4.21	2	2.11
Hallock; 2020	Partial repair	38	38	1	2.63	1	2.63
Heuberer; 2015	Partial repair	22	22	0	0.00	1	4.55
Heuberer; 2015	Complete repair	23	23	1	4.35	1	4.35
Jung; 2022	Partial repair	63	63	3	4.76	2	3.17
Kim; 2021	Partial repair	57	57	2	3.51	0	0.00
Lacheta; 2020	SCR	22	22	0	0.00	1	4.55
Leow; 2020	Complete repair	42	42	0	0.00	1	2.38
Ma; 2019	Complete repair	71	71	0	0.00	0	0.00
Malahias; 2019	Complete repair	34	34	0	0.00	1	2.94
Malahias; 2019	Partial repair	30	30	0	0.00	0	0.00
Takayama; 2021	SCR	54	54	1	1.85	0	0.00
Van der Zwaal; 2012	Complete repair	31	31	1	3.23	0	0.00
Yang; 2021	Complete repair	20	20	1	5.00	0	0.00

Abbreviations: LHB, long head of the biceps graft; Min, minimum; *N*, number; NR, not reported; RTSA, reverse total shoulder arthroplasty; SCR, superior capsular reconstruction; TNF, tensor fascia lata.

Table 5 shows the different average number of patients (expressed as\%) which resorted to a prosthesis, grouped the type of primary intervention. The percentage of reverse total arthroplasty and revision different from reverse total arthroplasty were compared between complete repair, partial repair and superior capsular reconstruction groups.

**Table 5:** Progression to revision surgery per intervention.

Intervention	*N* of studies	*N* of Patients	*N* of Shoulders	Revisions to RTSA	% RTSA	Revisions no RTSA	% no RTSA	SD
Complete repair	7	329	329	3	0.912	3	0.912	0.023
Partial repair	6	300	305	10	3.279	6	1.967	0.021
SCR	3	101	101	1	0.990	2	1.980	0.011

Abbreviations: *N*, number; RTSA, reverse total shoulder arthroplasty; SCR, superior capsular reconstruction; SD, standard deviation.

Complete repair was performed in seven studies, for a total of 329 patients and shoulders. Partial repair was performed in six studies, on 300 patients and 305 shoulders. Superior capsular reconstruction was performed in three studies over 101 patients and shoulders.

The proportion of revisions to reverse total arthroplasty was, 0.91%, 3.28% and 0.99%, for complete repair, partial repair and superior capsular reconstruction, respectively. From the raw data is it possible to observe a greater need for revision to reverse total arthroplasty when the original intervention was a partial repair. The outcome for superior capsular reconstruction and Complete Repair is almost equivalent in terms of raw proportions.

**Figure 5:**
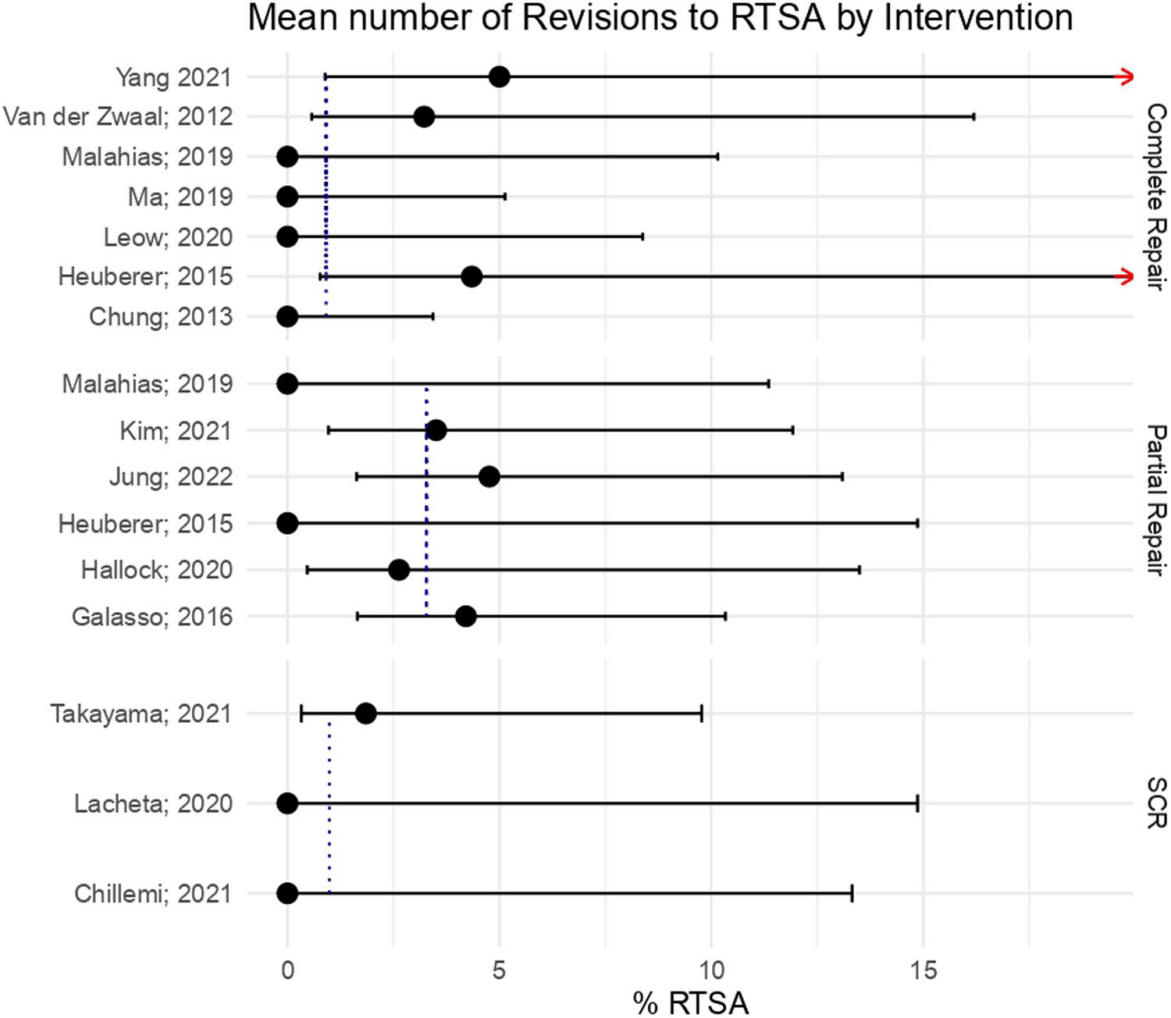
Mean number of revisions to reverse total shoulder arthroplasty (RTSA) by intervention.

However, no statistically significant differences were found across the groups in terms of progression to reverse total arthroplasty (n.s.) (Figure 5).

**Figure 6:**
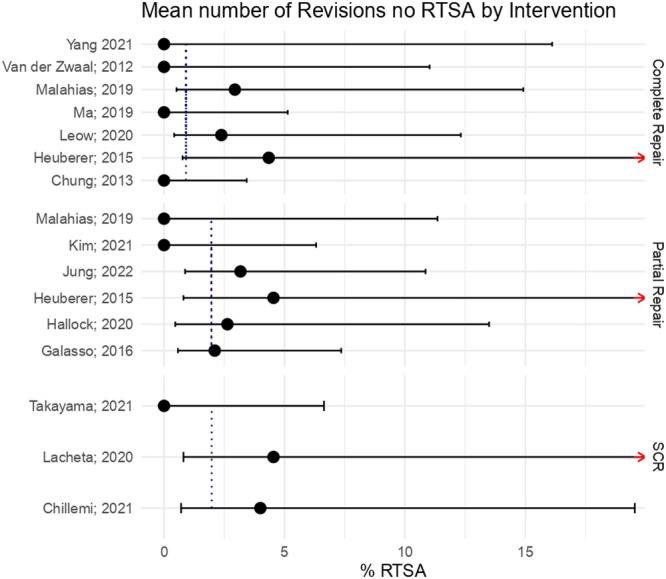
Mean number of revisions that did not progress to reverse total shoulder arthroplasty (RTSA) by intervention.

The average proportions of revisions to interventions different than reverse total arthroplasty were 0.91% for complete repair, 1.97% for partial repair and 1.98% for superior capsular reconstruction. In this case, the results are more similar across the Partial repair and superior capsular recontruction groups. However, once again there was no sufficient evidence to affirm that the average differs across groups with statistical significance (n.s.) (Figure 6).

The same analysis was done over the total number of revisions (reverse total arthroplasty or other than reverse total arthroplasty) needed after the primary surgery, divided by groups of intervention. Once again, no statistically relevant difference was found among the groups of intervention (n.s.). Data obtained from the meta‐analysis are summarised in Tables 4 and 5.

## DISCUSSION

The most important finding of the present study is that no statistically significant difference was found between partial repair, complete repair and superior capsular reconstruction in terms of progression to reverse total shoulder arthroplasty. Also, no statistically relevant differences were found among the groups of intervention in terms of progression to other revision procedures. There was, also, no difference in terms of total progression to either reverse total shoulder arthroplasty or other revision procedures. However, evidence of the raw data has demonstrated a greater number of patients progressing to reverse total shoulder arthroplasty following a partial repair compared to the other groups.

In the current literature there is lack of evidence on whether reverse total shoulder arthroplasty should be the primary surgery of choice for treatment of massive rotator cuff tears or whether it should be indicated as a secondary procedure after an arthroscopic attempt [6]. From this study, taking into consideration all included primary arthroscopic procedures, the rates of progression to reverse total shoulder arthroplasty were 0.9% in complete repair, 3.3% in partial repair and 0.1% in superior capsular reconstruction. These findings suggest that primary arthroscopic procedures may serve as a safe and viable treatment pathway in massive rotator cuff tear repair.

Seven studies performed complete repair for a total of 329 patients and shoulders, accounting for the lowest rate of progression to reverse total shoulder arthroplasty, as reported in the raw data. This surgical procedure presents a reliable treatment option for massive rotator cuff tears as it can be associated with good long‐term results and decelerated progression of joint degeneration [27]. Previous studies have shown significant improvement in pain and function whilst also providing high patient satisfaction and low retear rate accounting for 19%. Out of 31 patients undergoing complete repair, only one with a symptomatic re‐rupture underwent reverse shoulder arthroplasty. Despite this low progression to revision surgery, these results do not provide sufficient evidence given the relatively small sample size [52].

A complete anatomical repair is not always possible given that there are cases of chronic massive tendon retractions that are not feasible for this kind of treatment [12, 33]. Thus, implementation of a partial arthroscopic repair represents an appropriate alternative and has been suggested to reduce pain and improve functional outcomes. Furthermore, it has proven to have better outcomes when compared to other arthroscopic procedures such as debridement [13]. The application of suture augmentation technology in addition to the single row repair for patients with massive rotator cuff tears has been investigated, showing a lower incidence of recurrent tears compared to the standard procedure as well as the lack of revision surgery.

This study, however, only focused on comparing two similar surgical procedures both pertaining to partial repairs [36]. Improved outcomes would be anticipated in a complete repair due to its enhanced coverage of the humeral head leading to improved functional outcomes and pain alleviation [24]. On the one hand, it has been shown that complete repair of massive rotator cuff tears was associated with lower retear rates and improved functional outcomes compared to patients treated with partial repair [23]. However, several studies seem to show no statistically significant difference between these two approaches. Supporting this rationale, it was reported that partial arthroscopic repair proved to be not statically significantly inferior to complete repair and showed no revision surgeries in the former group [12].

The current meta‐analysis showed a revision rate to reverse total shoulder arthroplasty of 0.9% in the complete repair over 3.3% in the partial repair. Despite the raw data showing a greater need for revision surgery when the primary surgical intervention was partial repair, there is no sufficient evidence to affirm that the average differs across groups with statistical significance.

The present study also focused on analysing superior capsular reconstruction in the treatment for massive rotator cuff tears. This type of procedure has shown positive results, including restoring glenohumeral kinematics and humeral head translation, improvement in the range of motion and patient reported outcomes [1]. This surgical technique has then been modified using a dermal allograft to prevent donor site morbidity, a potential technique that has also been explored others [29]. In their study on superior capsular reconstruction procedures, it was found that utilising a dermal allograft led to significant improvements in clinical outcomes associated with high patient satisfaction as well as overall improvements, compared to reverse total shoulder arthroplasty. in the return‐to‐sport rate of young patients, thus showing a promising alternative in the treatment of massive rotator cuff tears.

According to the raw data of the current study, the outcome for superior capsular reconstruction and complete repair is almost equivalent in terms of raw proportions when analysing the rate of revisions to reverse total shoulder arthroplasty, however there are no statistically significant differences across the groups.

It is important to remember that arthroscopic treatment does not compromise subsequent reverse shoulder arthroplasty if this is needed [25] and the potential impact of previous rotator cuff repair surgery on reverse shoulder arthroplasty outcomes should be considered in future studies for counselling patients and identifying the highest value surgery for elderly patients with large tears. This has been previously investigated, stating that large dataset outcomes after reverse shoulder arthroplasty for rotator cuff repair such as cost, post‐acute care discharge, physical rehabilitation, and readmission rates appear not to be negatively affected by the presence of a prior rotator cuff repair [47].

Reverse shoulder arthroplasty has been also recognised as a primary treatment option for massive rotator cuff tears, rather than being treated as a revision surgery like in the previously mentioned studies. Despite reverse total shoulder arthroplasty being used primarily in elderly populations, indications for this therapeutic approach have been expanded to younger patients as well. Studies have shown significant improvement in pain relief, function and patient satisfaction among patients aged under 60 [47] or 65 years [19]. Despite these optimistic outcomes, there are notably high rates of complications, thus suggesting the need for alternative treatments. Given a 10 year survivorship of 58% in patients undergoing reverse total shoulder arthroplasty, studies have limited the recommendation for this approach to patients aged over 70 years with low functional demands [21]. This recommendation aligns with other studies [15] who noted clinical and radiological deterioration over time as well as lower rates of return‐to‐sport in patients who underwent reverse total shoulder arthroplasty and were younger than 65 years, emphasising caution in considering reverse shoulder arthroplasty as a primary approach for massive rotator cuff tears [39, 42]. Considering the apprehensions regarding the complications and long‐term outcomes following reverse total shoulder arthroplasty in young patients, minimally invasive and anatomy preserving techniques are most chosen as the primary approach. However, it remains uncertain whether the outcomes of these arthroscopic procedures are comparable or superior to those of reverse total shoulder arthroplasty and if there is an actual clinical benefit in repairing the tear with an initial less invasive approach, rather than moving straight to an arthroplasty.

The strength of this systematic review with meta‐analysis lies in the homogeneity of its population: only patients with massive rotator cuff tears were included. Patients diagnosed with large‐ or medium‐sized tears were not included in the cohort. Furthermore, no patients reporting pre‐operative severe shoulder osteoarthritis according to the Hamada classification were included, nor were those with irreparable subscapularis tears. Also, a minimum mean follow‐up of 12 months was required and only patients undergoing primary repair were selected.

Additionally, to improve the quality of the article, a meta‐analysis was performed, and all the included articles were subjectively evaluated by the Cochrane risk of Bias tools [48, 49] and by the critical appraisal tool by the Joanna Briggs Institute [43] in order to determine their potential risk of bias: articles judged as having critical or serious risk of bias were not included in the meta‐analysis.

The major limitation of this review lies in the lack of high‐quality randomised control trials: only one randomised control trial was available for the analysis, suggesting an impeding need for update on the topic. Furthermore, the rates of progression to reverse total shoulder arthroplasty were considered only for patients who underwent either complete repair, partial repair or superior capsular reconstruction as arthroscopic procedures of choice. This was due to the lack of studies reporting the use of other arthroscopic procedures, such as tendon transfers or balloon spacers, as a primary approach for massive rotator cuff tears.

Also, within the superior capsular reconstruction group, different surgical procedures were included: one article reported the use of a dermal allograft, another involved the use of an autograft from the Tensor Fascia Lata, and one exploited the long head of the biceps as an augmentation technique. This provides heterogeneity to the cohort and limits the validity of our findings.

## CONCLUSIONS

The current review finds no significant differences in the progression to reverse total shoulder arthroplasty or other revision procedures among partial repair, complete repair, and superior capsular reconstruction for massive irreparable rotator cuff tears. The absence of distinctions suggests comparable efficacy across interventions.

There is no consensus on the most effective treatment of massive rotator cuff tears due to the absence of high‐quality studies comparing arthroscopic procedures in terms of functional outcomes and revision rates. The effects of a prior failed arthroscopic rotator cuff repair on secondary reverse total shoulder arthroplasty remain yet to be investigated, and the benefits of reverse total shoulder arthroplasty as a primary procedure for massive rotator cuff tears are still unclear.

## AUTHOR CONTRIBUTIONS

*Conceptualization*: Umile Giuseppe Longo. *Methodology*: Benedetta Bandini and Alberto Lalli. *Software and validation*: Umile Giuseppe Longo. *Formal analysis*: Alice Piccolomini and Nathan S. Ullman. *Investigation*: Benedetta Bandini and Alberto Lalli. *Data curation*: Andrea Vaiano and Alice Piccolomini. *Writing—original draft preparation*: Nathan S. Ullman and Alice Piccolomini. *Writing—review and editing*: Umile Giuseppe Longo. *Visualization*: Pieter D'Hooghe. *Supervision*: Umile Giuseppe Longo. *Project administration*: Umile Giuseppe Longo and Pieter D'Hooghe. All authors have read and agreed to the published version of the manuscript.

## CONFLICT OF INTEREST STATEMENT

The authors declare no conflicts of interest.

## ETHICS STATEMENT

The Institutional Review Board of Campus Bio‐Medico University of Rome ruled that no formal ethics approval was required in this particular case.

## Data Availability

All relevant data analysed in this study is included in this published article and its supplementary information files. Further inquiries can be directed to the corresponding author.
